# When Professional Success Masks Neurodevelopmental Disorder: Late-Diagnosed ADHD in a Physician

**DOI:** 10.7759/cureus.107189

**Published:** 2026-04-16

**Authors:** Stephanie R Young

**Affiliations:** 1 Medicine/Medicine-Pediatrics, University of California Los Angeles, Los Angeles, USA

**Keywords:** adult attention deficit hyperactivity disorder (adhd), cognitive compensation, comorbid depression, neuropsychology assessment, physician mental health

## Abstract

Attention-deficit/hyperactivity disorder (ADHD) is increasingly recognized as a lifespan neurodevelopmental condition that persists into adulthood in a substantial proportion of individuals. Despite growing awareness, adult ADHD is frequently underrecognized, particularly in those with high intellectual ability and sustained academic or professional success. Misconceptions that high achievement without external supports excludes ADHD can delay diagnosis. This case describes a physician in his 30s with lifelong inattentive symptoms and inefficiency, who excelled in patient care yet struggled with selective task organization and completion. Years earlier, his difficulties were attributed to depression, likely influenced by the demands of medical training. When attentional symptoms persisted and began to affect personal relationships, he underwent a comprehensive neuropsychological evaluation. Testing revealed selective attentional and executive functioning vulnerabilities consistent with ADHD, Predominantly Inattentive Presentation. Depressive symptoms, including guilt and self-blame, were secondary to repeated lapses in attention and incomplete task follow-through. Receiving a formal ADHD diagnosis was profoundly clarifying, allowing the patient to pursue targeted interventions and better understand the relationship between chronic inefficiencies and self-perception. This case illustrates the value of neuropsychological assessment in clarifying diagnosis when achievement history may mask symptoms or when depressive features appear prominent. Recognition of ADHD in high-functioning adults has implications for timely intervention, self-awareness, and optimization of personal and professional functioning.

## Introduction

Attention-deficit/hyperactivity disorder (ADHD) is a neurodevelopmental condition that frequently persists into adulthood [1], with prevalence estimates ranging from 2.5% to 3.1% in adult populations [2,3]. In adults, ADHD often presents with symptoms of inattention, executive dysfunction, and difficulty sustaining effort, rather than overt hyperactivity [4]. Despite its prevalence and functional impact, ADHD remains underrecognized in adults, particularly among individuals with high levels of academic or professional achievement [5,6].

One challenge in identifying ADHD in adulthood is the reliance on external markers of functioning, such as educational attainment or occupational success, as proxies for intact attentional capacity. High-functioning individuals may compensate for attentional vulnerabilities through cognitive strengths, structured environments, and sustained effort, allowing them to meet external expectations while experiencing significant internal inefficiency [7]. As a result, functional impairment may not become clinically apparent until environmental supports decrease or demands exceed an individual’s capacity for compensation [8].

Medical training represents a uniquely structured and prolonged environment characterized by clear expectations, frequent evaluation, and strong external accountability. These features may facilitate compensation for attentional difficulties and delay recognition of ADHD [9,10]. At the same time, the assumption that untreated ADHD is incompatible with successful completion of medical training may contribute to diagnostic overshadowing, particularly when attentional symptoms overlap with mood-related concerns such as fatigue, low motivation, or reduced concentration [11].

Neuropsychological evaluation may be helpful in clarifying complex presentations of adult ADHD, particularly in high-functioning individuals. Objective assessment can identify patterns of cognitive strengths and relative inefficiencies that may not be apparent from academic history or professional performance alone. However, ADHD remains a clinical diagnosis based upon developmental history, cross-situational symptoms, and functional impairment, and neuropsychological findings are not specific to ADHD. Accordingly, such testing is best conceptualized as a tool that can support, but not establish, diagnostic formulation when symptom overlap exists [6,12].

This case report describes a physician diagnosed with ADHD in adulthood after a longstanding academic and professional success and a prior attribution of symptoms to depression. The case illustrates how compensatory strategies and environmental structure may mask ADHD across development, how diagnostic assumptions may delay recognition, and how integration of clinical history and neuropsychological data may support diagnostic clarification in select cases. It also highlights the potential value of involving family members in feedback to improve shared understanding and functional outcomes.

## Case presentation

The patient is a physician in his 30s who presented with a concern that attentional issues were increasingly impacting his ability to carry out professional and personal tasks that required self-directed organization and follow-through. Several years prior, he had presented to psychiatry with a 12-month history of depressed mood, negative self-talk, guilt, and occasional forgetfulness, with a lack of an identifiable trigger. He had reported that his academic functioning was impacted by these mood issues, as he was unable to efficiently complete administrative assignments and tasks, and he was diagnosed with Other Specified Depressive Disorder. He expressed that he was not sure that the prior diagnosis explained his variable forgetfulness, follow-through on tasks, and difficulty with time management. He was referred for a neuropsychological assessment to uncover whether there were areas of neurocognitive weakness that could be explanatory.

Clinical evaluation

During the neuropsychological clinical interview, the patient reported a longstanding pattern of inattention, including forgetfulness, difficulty organizing tasks, procrastination, inconsistent follow-through, and impaired time management. These symptoms were present across multiple contexts, including academic, occupational, and interpersonal settings.

Symptoms consistent with inattentive ADHD were reported prior to age 12 based on a detailed clinical interview, including a history of distractibility, difficulty sustaining attention during assignments, and teacher observations of inattentiveness without behavioral disruption. Although one retrospective self-report measure did not reach the cutoff threshold, the discrepancy was considered in interpretation, with greater weight placed on developmentally contextualized clinical history rather than questionnaire thresholds alone, consistent with DSM-5-TR criteria.

Across development, symptoms persisted but were partially mitigated by strong intellectual abilities, structured environments, and increasingly effortful compensatory strategies. He reported academic giftedness that allowed him to perform well throughout primary and secondary education without the need for special educational services or formal accommodations. His home environment was characterized by consistent oversight and support of his studies by his parents. Functional impairment became more apparent during transitions, specifically during the first year of high school and early college, when he encountered less structured environments. In college, he experienced greater difficulty sustaining attention, organizing academic work, and maintaining consistent performance, at times earning grades below his perceived ability. However, he adopted increasingly individualized study strategies, including constructing his own study materials, which allowed him to improve encoding and recall of information. These strategies were later applied successfully during medical school and standardized exam preparation. Over time, the nature of his symptoms evolved, with less overt distractibility and greater difficulty with time management, task initiation, and sustained effort.

At the time of the evaluation, he reported difficulty with non-patient care tasks requiring self-directed organization and follow-through, with prominent procrastination and inconsistent task completion. What was increasingly bothersome were similar patterns at home, where he frequently forgot agreed-upon tasks and had difficulty initiating and planning activities with his spouse, leading to more significant relational difficulties. He noted comparable challenges in maintaining social commitments with friends. These difficulties had been present for several years.

Neuropsychological findings

Intellectual functioning was assessed using the Wechsler Adult Intelligence Scale-Fourth Edition [13]. Attention and vigilance were evaluated using the Conners Continuous Performance Test, Third Edition [14]. Executive functioning was assessed using selected subtests from the Delis-Kaplan Executive Function System [15]. Verbal learning and memory were evaluated using the California Verbal Learning Test-Second Edition [16], and additional memory functions were assessed using the Wechsler Memory Scale-Fourth Edition [17]. Emotional functioning was assessed using the Beck Depression Inventory-Second Edition [18] and the Beck Anxiety Inventory [19].

Formal neuropsychological testing demonstrated superior overall intellectual functioning on the Wechsler Adult Intelligence Scale-Fourth Edition (Full Scale IQ = 121; 92nd percentile), with particularly strong verbal abilities (Verbal Comprehension Index = 130; 98th percentile). Working memory and processing speed were in the high average range (Working Memory Index = 111; 77th percentile; Processing Speed Index = 117; 87th percentile). While these performances fall within normative limits and no index score revealed an impairment, working memory represented a relative intra-individual weakness compared to verbal reasoning abilities.

Performance across many executive functioning measures was intact or above average, indicating preserved higher-order executive abilities. However, subtle relative inefficiencies were observed in sustained attention and learning.

On a continuous performance task (Conners Continuous Performance Test, Third Edition), the patient demonstrated increased commission errors and variability under conditions of reduced stimulus frequency, consistent with subtle difficulties sustaining attention and vigilance over time. On a verbal learning task (California Verbal Learning Test, Second Edition), overall acquisition across repeated trials was consistent with his intellectual ability, but elevated repetitions and slightly lower delayed recall suggested reduced efficiency in monitoring and consolidating information, rather than a primary memory deficit. While similar patterns can be seen in various conditions, in this context, these results were interpreted as indicative of attentional inefficiency impacting learning and retrieval processes.

On emotional assessment, the patient endorsed minimal symptoms on the Beck Depression Inventory-Second Edition and Beck Anxiety Inventory. He endorsed mild, transient symptoms of self-criticism and guilt, which he attributed to perceived underperformance and concern about disappointing others.

Importantly, these results do not represent diagnostic markers of ADHD. Rather, they illustrate a pattern of relative weaknesses and intra-individual variability, which were considered alongside clinical history and functional impairment in diagnostic formulation.

A summary of neuropsychological test performance is presented in Table 1.

**Table 1:** Summary of Neuropsychological Test Performance With Clinical Interpretation Both statistical scores and brief functional interpretations to facilitate clinical understanding are included. Scores between the 25th and 75th percentiles are generally considered within the average range, while scores below the 16th percentile may reflect clinically significant weakness. Scores above the 84th percentile are considered above average. In this table, the term relative weakness indicates performance that is lower than expected for this individual’s cognitive ability and may correspond to areas of inefficiency in real-world functioning (e.g., inconsistent attention, difficulty completing tasks efficiently, or problems with learning new information), even when scores fall within normative limits. Scores are normed using standard normative samples. T-scores have a mean of 50 and standard deviation of 10. WAIS-IV Index Scores have a mean of 100 and a standard deviation of 15; WAIS-IV Subtest Scaled Scores have a mean of 10 and a standard deviation of 3. Z-scores have a mean of 0 and a standard deviation of 1. Raw scores are provided for clarity and context; percentiles are based on normative data. Interpretations consider both absolute performance and relative expectations based on the patient’s overall intellectual ability. All results should be interpreted in the context of the individual’s overall cognitive profile and clinical history, as isolated scores are not diagnostic of any condition. WAIS-IV: Wechsler Adult Intelligence Scale-Fourth Edition; FSIQ: Full Scale IQ; CPT-3: Conners Continuous Performance Test, Third Edition; CVLT-II: California Verbal Learning Test-Second Edition; BDI-II: Beck Depression Inventory-Second Edition; BAI: Beck Anxiety Inventory

Domain	Measure	Score	Percentile	Interpretation
Intellectual Functioning	WAIS-IV FSIQ	SS = 121	92nd	Superior
	Verbal Comprehension Index	SS = 130	98th	Very superior
	Working Memory Index	SS = 111	77th	High average (relative weakness)
	Processing Speed Index	SS = 117	87th	High average
Attention	Digit Span	SS = 13	84th	High average; qualitatively effortful
	CPT-3 Detectability (d’)	T = 60	84th	Elevated (reduced discrimination; difficulty distinguishing target vs. non-target stimuli)
	CPT-3 Commissions	T = 67	95th	Elevated (impulsive response style; tendency to respond quickly with errors)
	CPT-3 ISI Change	T = 64	92nd	Elevated (reduced consistency over time; difficulty sustaining attention across tasks)
Learning & Memory	CVLT-II Trials 1-5 Total	T = 48	42nd	Average acquisition across repeated exposures
	CVLT-II Learning Slope	Raw = 1.5	50th	Average rate of acquisition
	CVLT-II Long Delay Free Recall	Raw = 9	16th	Low average (less efficient retrieval relative to overall ability)
	CVLT-II Repetitions	Raw = 14	1st	Impaired (inefficient encoding; reduced self-monitoring)
Executive Functioning	Trail Making Switching	SS = 13	84th	High average
	Letter Fluency	SS = 17	99th	Superior
Emotional Functioning	BDI-II	8	-	Minimal
	BAI	2	-	Minimal

Diagnostic formulation

Diagnostic formulation was guided by DSM-5-TR criteria, including the presence of multiple inattentive symptoms across development, persistence into adulthood, and clinically significant impairment across settings. Impairment was evident in occupational (non-clinical responsibilities), interpersonal (spousal relationship), and daily functioning domains. Observations from the patient’s spouse further supported the presence of cross-situational attentional difficulties.

Alternative explanations for attentional inefficiency were systematically evaluated. The patient denied persistent symptoms consistent with major depressive disorder or an anxiety disorder and endorsed minimal symptoms on standardized measures at the time of assessment. Feelings of guilt, self-criticism, and disappointment were temporally linked to perceived underperformance and feedback from close others regarding his lack of follow-through, suggesting they were more likely secondary to attentional challenges rather than indicative of a primary mood disorder. Although he reported periods of fatigue and burnout during medical training, these were described as situational and transient rather than pervasive. Sleep disruption, substance use, and other medical or psychiatric contributors to attentional dysfunction were assessed through clinical interview and were not supported as primary or sufficient explanations for the patient’s longstanding symptoms. While such factors can contribute to attentional variability, the chronic, developmentally consistent nature of the patient’s symptoms and the pattern of functional impairment were considered more consistent with a neurodevelopmental etiology. Therefore, the patient’s presentation was felt to be most consistent with ADHD, Predominantly Inattentive Presentation, when considered in the context of developmental history, functional impairment, and integrated clinical assessment.

Neuropsychological feedback

Following a comprehensive evaluation, a detailed feedback session was conducted with the patient and his spouse. Recommendations included psychoeducation on adult ADHD to enhance understanding of symptoms and functional impact, discussion of pharmacologic treatment options, and implementation of cognitive strategies to support learning, organization, planning, and time management. Behavioral interventions included cognitive-behavioral therapy (CBT) tailored for ADHD, executive function coaching, and self-monitoring of strategy use to reinforce skill acquisition. Emphasis was placed on specific communication strategies and maintaining a consistent daily schedule to mitigate symptom-related disruptions. Additionally, curated resources were provided, including websites, community support programs, and literature on adult ADHD, to facilitate ongoing self-education and skill development.

Importantly, the feedback session included discussion of specific communication strategies for both the patient and his spouse (e.g., establishing eye contact before communicating, brief repetition or summarization of requests, and minimizing competing distractions). These strategies were intended to support attentional engagement during conversations and improve follow-through on shared responsibilities. Facilitating this shared understanding of attentional vulnerabilities allowed the couple to collaboratively implement practical adjustments in daily communication, with the goal of reducing frustration and improving quality of life. Involving both partners in the feedback session also promoted a shared framework for understanding the diagnosis and encouraged collaborative implementation of behavioral strategies in both personal and professional contexts.

Receiving the ADHD diagnosis was profoundly clarifying and relieving for the patient. It allowed him to contextualize his longstanding attentional lapses, reduce self-blame, and begin implementing strategies to improve daily functioning. The diagnosis also had a significant impact on his spouse, who had previously interpreted his behaviors as deliberate inattention or lack of engagement. Understanding the neurodevelopmental basis of these difficulties reframed these behaviors, fostering greater empathy and improving collaboration in managing household and personal responsibilities. Follow-up data after the feedback session were not available for this case.

## Discussion

This case illustrates how ADHD may remain unrecognized in high-functioning adults due to prolonged compensation, preserved cognitive strengths, and assumptions based on academic and professional success. Although ADHD is increasingly recognized as a lifespan neurodevelopmental condition, diagnostic consideration in adults often relies heavily on external markers of functioning, which may obscure underlying attentional vulnerabilities.

Compensation and delayed recognition in high-functioning adults

The patient described demonstrated sustained academic and professional achievement without formal accommodations, a history that may be interpreted as inconsistent with ADHD. However, functional success does not preclude underlying neurodevelopmental vulnerability. In structured environments with clear expectations and external accountability, individuals with ADHD may rely on compensatory strategies (i.e., excessive effort, self-imposed structure, and cognitive strengths) to meet performance demands [20]. Over time, particularly as environments become less structured and responsibilities more complex, these strategies may become insufficient, leading to increased inefficiency and subjective impairment [8].

Medical training represents a prolonged period of externally imposed structure, frequent evaluation, and clear performance metrics. These features may facilitate compensation for attentional difficulties and delay recognition of ADHD. The physician identity in this case serves to highlight how diagnostic assumptions regarding professional success can contribute to underrecognition of ADHD.

Selective executive dysfunction and the role of neuropsychological evaluation

This case illustrates how ADHD may remain unrecognized in individuals with superior intellectual functioning, as cognitive strengths and compensatory strategies can obscure attentional vulnerabilities. Neuropsychological evaluation in this case identified subtle intra-individual variability, with performance generally within normative limits but below expectation relative to intellectual ability in domains such as sustained attention, inhibitory control, and learning efficiency. While subtle, these discrepancies are clinically meaningful and may not have been captured through clinical interview alone in all cases, increasing the risk of underdiagnosis or misinterpretation of symptoms.

Importantly, these findings may be observed in other conditions, including stress, sleep disruption, mood symptoms, or burnout, and thus are not specific to ADHD. Accordingly, neuropsychological data were not used to establish the diagnosis independently, but rather to contextualize the patient’s reported difficulties and support clinical interpretation.

This pattern highlights the heterogeneity of executive functioning in ADHD and challenges the assumption that the disorder is uniformly associated with global executive dysfunction. Current models of ADHD emphasize variability across executive domains, with some individuals showing intact executive abilities alongside impairments in sustained attention and vigilance [21]. In high-functioning adults, preserved executive abilities may further mask impairments in sustained attention and consistency, particularly when evaluation relies primarily on educational functioning or occupational attainment [22].

Neuropsychological testing may therefore be most useful in complex or ambiguous cases as an adjunctive tool, particularly when there is discordance between reported symptoms and observed functioning. Careful integration of test findings with developmental history and real-world impairment remains essential.

Diagnostic considerations and differential diagnosis

This case also underscores the importance of careful differential diagnosis in adults presenting with attentional concerns. Symptoms such as forgetfulness, inefficiency, and reduced concentration may arise from multiple causes, including mood disorders, anxiety, burnout, sleep disturbance, and situational stress.

In this case, alternative explanations were systematically evaluated through detailed clinical interview and were not supported as primary drivers of symptoms. Mood-related symptoms were intermittent, context-dependent, and closely linked to functional challenges, rather than persistent or pervasive. While such distinctions may not always be definitive, the overall pattern was most consistent with a neurodevelopmental attentional disorder. Nevertheless, it is important to acknowledge that retrospective assessment of childhood symptoms is inherently limited, and that diagnostic conclusions in adult ADHD often involve some degree of clinical inference.

Diagnostic overshadowing

The patient’s attentional concerns were initially attributed to depression, reflecting a common diagnostic pathway given overlapping symptoms of poor concentration and reduced motivation. In this case, depressive features appeared more consistent with secondary emotional responses to chronic inefficiency and underperformance, although causality cannot be established definitively. Instead of being reflective of a depressive disorder, self-blame, guilt, and fear of disappointing others may reflect performance failures associated with untreated ADHD [23]. This highlights the importance of reassessing diagnostic formulations when symptoms persist despite initial explanations.

Clinical implications for Med-Peds practice

For Med-Peds clinicians, this case emphasizes the value of a developmental approach to attentional symptoms and illustrates how assumptions linking professional achievement with intact attentional functioning may delay recognition of ADHD in highly accomplished adults. ADHD should not be excluded solely on the basis of academic or professional success. Instead, careful attention to symptom persistence across contexts, the effort required to maintain functioning, and the developmental evolution of compensatory strategies may improve diagnostic accuracy. When clinical presentation is complex or atypical, neuropsychological evaluation may serve as a useful adjunct to support diagnosis and guide treatment. Additionally, involving family members in the feedback process may be particularly valuable in adult ADHD, as shared understanding of attentional vulnerabilities can reduce interpersonal misattributions (e.g., interpreting symptoms as a lack of effort or engagement) and support collaborative implementation of behavioral strategies.

## Conclusions

ADHD may remain unrecognized in high-functioning adults when cognitive strengths, structured environments, and assumptions about professional competence obscure underlying attentional vulnerabilities. This case illustrates how prolonged reliance on compensatory strategies can allow individuals with ADHD to achieve substantial academic and professional milestones, including completing medical training, while masking inefficiencies in attention and executive regulation. This case suggests that clinicians should be cautious about excluding ADHD on the basis of academic or occupational success. In complex or atypical presentations, neuropsychological evaluation may provide useful adjunctive information by identifying patterns of relative strengths and weaknesses. While neuropsychological findings are not highly specific for ADHD, they may meaningfully contribute to diagnostic formulation in the broader clinical context. Diagnostic conclusions should be based on integration of developmental history, cross-contextual symptoms, and functional impairment, with neuropsychological data serving a supportive role.

Importantly, diagnostic clarification may also facilitate shared understanding within close relationships, reducing misinterpretation of symptoms and supporting collaborative management strategies. In this case, inclusion of the patient’s spouse in feedback appeared to enhance collaboration, highlighting a potential avenue for improving functional outcomes in similar cases. Finally, collaboration between neuropsychologists, the patient, the family, and the primary care provider supports patient insight into the diagnosis, reframes understanding of longstanding attentional concerns, and enables engagement with personalized treatment planning to improve long-term functioning and quality of life.
